# Comparison of alternative approaches for difference, noninferiority, and equivalence testing of normal percentiles

**DOI:** 10.1186/s12874-020-00933-z

**Published:** 2020-03-13

**Authors:** Gwowen Shieh

**Affiliations:** Department of Management Science, National Chiao Tung University, Hsinchu, Taiwan 30010 Republic of China

**Keywords:** Power, Quantile, Reference limit, Sample size

## Abstract

**Background:**

Percentiles are widely used in scientific research for determining the comparative magnitude and reference limit of quantitative measurements. The investigations for point and interval estimation of normal percentiles are well documented in the literature. However, the corresponding statistical tests of hypothesis have received relatively little attention.

**Methods:**

To facilitate data analysis and design planning of percentile study, this paper aims to present hypothesis testing procedures and associated power functions for assessing the difference, noninferiority, and equivalence of normal percentiles.

**Results:**

Numerical illustrations about drug dissolution are provided to demonstrate the usefulness of the suggested exact approaches and the deficiency of approximate methods.

**Conclusions:**

The exact approaches are superior to the approximate methods on the basis of control of Type I errors. Computer algorithms are constructed to implement the recommended test procedures and sample size calculations for percentile analysis.

## Background

Percentiles are extremely useful for describing the reference threshold and meaningful magnitude of numerical quantities, such as achievement score, developmental index, medical measurement, and physical dimension. The inferential methods for normal means are well documented in the fundamental texts of statistical analysis. However, the methodological aspects and statistical implications of analyzing normal percentiles have been less discussed. It is essential to note that normal percentiles are a linear function of the mean and standard deviation of the underlying population. Because the sample mean and sample variance are complete and sufficient statistics for the population mean and variance, the minimum variance unbiased estimator of a normal percentile can be readily obtained. Specifically, Royston and Mathews [1] compared the minimum variance unbiased estimator and other useful formulas under the intrinsic criteria of bias and mean square error. More advanced and theoretical treatments of normal percentile estimation are also available in Keating, Mason, and Balakrishnan [2], Keating and Tripathi [3], Parrish [4], Rukhin [5], and Zidek [6, 7].

Both exact and approximate confidence intervals of normal percentiles have been considered in several analytical developments. The exact interval estimation of normal percentiles was presented in Meeker, Hahn, and Escobar [8], Johnson, Kotz, and Balakrishnan [9], and Owen [10]. Note that the exact confidence intervals involve the quantiles of a noncentral *t* distribution. Such critical values are not commonly available in tabulated forms and the implementation necessitates appropriate computing algorithms. To circumvent the reliance on a noncentral *t* distribution, approximate methods were considered by using the standardization technique and the regular *t* distribution. Accordingly, the approximate confidence intervals of Bland and Altman [11] and Chakraborti and Li [12] are computationally simple and the interval calculations do not require specialized software. However, the numerical study of Shieh [13] demonstrated that the confidence limits of the approximate methods generally do not preserve the nominal equal-tailed error rates. The finding provides cautionary counterpoint on the practical value of approximate intervals, especially when the sample sizes are small.

The existing investigations present important inferential methodology for point and interval estimation of normal percentiles. However, the related hypothesis testing problems have not been properly explicated in the literature. It is well known that there exists a direct connection between confidence interval and hypothesis testing. But the two approaches are philosophically different in the outset of precision and power viewpoints. Accordingly, to conduct a significance tests for percentiles, the conclusion can be alternatively obtained by examining whether the specified percentile value is contained in the proper two- or one-sided confidence intervals. It appears that percentile analysis can be performed without explicitly defining the desirable test statistics and associated rejection regions. However, power evaluation and sample size planning for hypothesis testing methodologically differ from the precision and sample size considerations in the context of interval estimation. Consequently, it is of theoretical importance and practical interest to document the exact test procedures, power calculations, and sample size determinations for percentile studies.

To enhance the usage of percentile analysis, this article describes hypothesis testing procedures and associated power functions for assessing the difference, noninferiority, and equivalence of normal percentiles. The difference and noninferiority procedures closely follow the two- and one-tail test formulations. In the conventional studies of the population means, a null hypothesis of zero may be informative to address certain essential research questions. The situations associated with percentile assessment are more sophisticated because the target percentile is unlikely a zero value. The percentile tests for difference and noninferiority require researchers to provide a sensible magnitude that corresponds to the percentile threshold for identifying substantial research finding. Moreover, the importance for establishing equivalence instead of no difference has been emphasized in Blackwelder [14] and Parkhurst [15], among others. Further details on the design and analysis of noninferiority and equivalence studies can be found in Fleming et al. [16] and Wellek [17].

Notably, the binomial test of hypotheses concerning quantiles in Mood, Graybill, and Boes ([18], Section 11.3.2) provides an appealing nonparametric alternative. Although the procedure is applicable for all random samples from a continuous distribution, there are not many feasible alpha values for small sample sizes, unless randomized tests are used. In general, the nonparametric tests may be more powerful than their parametric counterparts when normality assumption fails, whereas the nonparametric alternatives are less powerful than the parametric procedures when the conventional assumptions hold. More importantly, the undesirable properties and related problems associated with binomial tests have been addressed in Vos and Hudson [19] and Thulin [20], among others. Comprehensive discussions and reviews for the prevailing Wald large-sample normal test and other alternative interval procedures can be found in Agresti and Coull [21], Newcombe [22], Brown, Cai, and DasGupta [23, 24], and the references therein. The illustrations and appraisals in this article were confined to the test procedures that assume normality of the sampling distribution.

This paper aims to present the exact test procedures for percentile study under the three structural considerations of difference, noninferiority, and equivalence scenarios. For the purpose of providing profound implications in selecting the most appropriate approach, the approximate techniques of Bland and Altman [11] and Chakraborti and Li [12] are also extended to the percentile testing problem. Specifically, Bland and Altman [11] proposed an approximate *t* distribution for a convenient transformation of the natural, but biased, estimator of the normal percentile. On the other hand, Chakraborti and Li [12] suggested that a standardized minimum variance unbiased estimator also has an approximate *t* distribution. Note that the simplified considerations proposed in Bland and Altman [11] and Chakraborti and Li [12] may be appealing for inducing computational shortcuts but they do not necessarily maintain the desired accuracy for all settings, especially when the sample sizes are small. Accordingly, it is essential to discern not only which method is most suitable under what circumstances but also the actual differences between the contending test procedures.

Furthermore, the corresponding power and sample size calculations for advance planning of percentile studies are explicated. Monte Carlo simulation study was also conducted to compare the accuracy of the exact and approximate procedures with respect to the control of Type I error rate. Although an exact technique is theoretically better than the approximate methods, the actual performance may not guarantee a substantial difference to justify the need for adopting the exact approach that is methodologically sophisticated and computationally demanding. The current study provides detailed analytic explications and numerical evidences to reveal the discrepancy between the exact and approximate procedures for percentile analysis. A drug dissolution problem and accompanying software programs are employed to illustrate the usefulness of suggested procedures for data analysis and design planning.

## Methods

### Exact test procedures

Assume *X*_1_, …, *X*_*N*_ are a sample from a *N*(μ, σ^2^) population with unknown mean μ and variance σ^2^ for *N* > 1. The 100*p*th percentile of the normal distribution *N*(μ, σ^2^) is denoted by θ, where

1 $$ \uptheta =\upmu +{\mathrm{z}}_{\mathrm{p}}\upsigma $$

and *z*_*p*_ is the (100·*p*)th percentile of the standard normal distribution *N*(0, 1). An intuitive, but biased, estimator of the percentile θ is

2 $$ {\hat{\theta}}_B=\overline{X}+{z}_pS, $$

where $\overline{X}=\sum \limits_{i=1}^N{X}_i/N$ and ${S}^2=\sum \limits_{i=1}^N{\left({X}_i-\overline{X}\right)}^2/\left(N-1\right)$ are the sample mean and sample variance, respectively. Accordingly, the minimum variance unbiased estimator is

3 $$ {\hat{\theta}}_M=\overline{X}+{z}_p cS. $$

where *c* = (ν/2)^1/2^Γ(ν/2)/Γ{(ν + 1)/2} and ν = *N* – 1. Further details about the point estimation properties of ${\hat{\theta}}_B$ and ${\hat{\theta}}_{MU}$ are available in Royston and Mathews [1]. Also, the recent study of Shieh [13] compared several confidence interval procedures of θ. In contrast, the focus here is on the hypothesis testing of normal percentiles.

Under the prescribed normal setting for the sample {*X*_1_, …, *X*_*N*_}, standard derivations show that

4 $$ {T}_E=\frac{\overline{X}-\theta }{{\left({S}^2/N\right)}^{1/2}}\sim t\left(v,-{z}_p{N}^{1/2}\right), $$

where *t*(ν, −*z*_*p*_*N*^1/2^) is a noncentral *t* distribution with degrees of freedom ν and noncentrality parameter –*z*_*p*_*N*^1/2^. The fundamental properties and related extensions of noncentral *t* distribution can be found in Johnson, Kotz, and Balakrishnan [9].

### Tests for difference

To detect the magnitude of a percentile in terms of the hypotheses

5 $$ {\mathrm{H}}_0:\uptheta ={\uptheta}_0\ \mathrm{versus}\ {\mathrm{H}}_1:\uptheta \ne {\uptheta}_0, $$

the test statistic is of the form

6 $$ {T}_{E0}=\frac{\overline{X}-{\uptheta}_0}{{\left({S}^2/N\right)}^{1/2}}, $$

where θ_0_ is a constant. The test rejects H_0_ at the significance level α if *T*_*E*0_ < τ_α/2_ or *T*_*E*0_ > τ_1 − α/2_ where τ_α/2_ and τ_1 − α/2_ are the lower and upper (100·α/2)th quantiles of the distribution *t*(ν, −*z*_*p*_*N*^1/2^), respectively, for 0 < α < 0.5. Accordingly, it can be shown that the power function is of the form

7 $$ {\varPsi}_{DI}\left(\varDelta \right)=P\left\{t\left(v,\varDelta \right)<{\uptau}_{\upalpha /2}\right\}+P\left\{t\left(v,\varDelta \right)>{\uptau}_{1-\upalpha /2}\right\}, $$

where Δ = (μ – θ_0_)/(σ^2^/*N*)^1/2^.

### Tests for noninferiority

In addition to the regular test of difference, it is of practical importance to test the hypotheses for noninferiority. The problem of testing noninferiority of percentiles can be presented by the following hypotheses:

8 $$ {\mathrm{H}}_0:\uptheta \le {\uptheta}_0\;\mathrm{versus}\ {\mathrm{H}}_1:\uptheta >{\uptheta}_0 $$

when larger values of θ are desired and θ_0_ is the designated noninferiority threshold. The test procedure rejects the null hypothesis at the significance level α if *T*_*E*0_ > τ_1 − α_ and the associated power function is readily obtained as

9 $$ {\varPsi}_{NI}\left(\varDelta \right)=P\left\{t\left(v,\varDelta \right)>{\uptau}_{1-\upalpha}\right\}. $$

On the other hand, if smaller values of θ are preferred, then the following hypotheses should be adopted for the test of noninferiority:

10 $$ {\mathrm{H}}_0:\uptheta \ge {\uptheta}_0\mathrm{versus}\ {\mathrm{H}}_1:\uptheta <{\uptheta}_0, $$

where the chosen value θ_0_ represents the noninferiority bound. At the significance level α, the rejection region for the lower one-sided test is *T*_*E*0_ < τ_α_ and the power function is expressed as

11 $$ {\varPsi}_{NI}\left(\varDelta \right)=P\left\{t\left(v,\varDelta \right)<{\uptau}_{\upalpha}\right\}. $$

### Tests for equivalence

Unlike the traditional differences-based procedures, equivalence testing provides a proper method for demonstrating the comparability of target percentile. In general, the null and alternative hypotheses of a test of percentile equivalence can be formulated as

12 $$ {\mathrm{H}}_0:\uptheta -{\uptheta}_T\le -\updelta\;\mathrm{or}\;\uptheta -{\uptheta}_T\ge \updelta\;\mathrm{versus}\ {\mathrm{H}}_1:-\updelta <\uptheta -{\uptheta}_T<\updelta, $$

where θ_*T*_ and δ (> 0) are constants. Accordingly, θ_*T*_ is the target value and δ represents the minimum threshold for declaring equivalence between the population percentile θ and θ_*T*_. Following the two one-sided tests procedure proposed by Schuirmann [25] and Westlake [26] for assessing equivalence of mean effects, the null hypothesis is rejected at the significance level α if

13 $$ {T}_{EL}=\frac{\overline{X}-{\uptheta}_T+\updelta}{{\left({S}^2/N\right)}^{1/2}}>{\uptau}_{1-\upalpha}\;\mathrm{and}\;{T}_{EU}=\frac{\overline{X}-{\uptheta}_T-\updelta}{{\left({S}^2/N\right)}^{1/2}}<{\uptau}_{\upalpha}. $$

It is important to note that the rejection is an intersection of two one-sided segments in terms of the lower and upper (100·α)th quantiles τ_α_ and τ_1 − α_ of the noncentral *t* distribution *t*(ν, −*z*_*p*_*N*^1/2^). The rejection region of $\overline{X}$ and *S*^2^/*N* has an isosceles triangular shape similar to those in Meyners [27] and Schuirmann [28] for the equivalence procedure of two treatment means. Consequently, the power function of the percentile equivalence test can be written as

14 $$ {\varPsi}_{EQ}=P\left\{{\uptheta}_T-\updelta +{\uptau}_{1-\upalpha}{\left({S}^2/N\right)}^{1/2}<\overline{X}<{\uptheta}_T+\updelta +{\uptau}_{\upalpha}{\left({S}^2/N\right)}^{1/2}\right\}. $$

Moreover, it is clear from the fundamental assumption in Eq. 1 that $Z=\left(\overline{X}-\upmu \right)/{\left({S}^2/N\right)}^{1/2}\sim N\left(0,1\right)$ and *K* = ν*S*^2^/σ^2^ ~ χ^2^(ν), where χ^2^(ν) denotes the chi-square distribution with ν = *N* – 1 degrees of freedom, and *Z* and *K* are independent. Let *H*_*E*_ = 1 if *K* < κ_*E*_, and *H*_*E*_ = 0 if *K* ≥ κ_*E*_ where *κ*_*E*_ = (4*vN*δ^2^)/{*σ*^2^(τ_1 − α_ − τ_α_)^2^}. Then, the exact power function can be expressed by

15 $$ {\varPsi}_{EQ}={E}_K\left[{H}_E\left\{\varPhi \left({U}_E\right)-\varPhi \left({L}_E\right)\right\}\right], $$

where *U*_*E*_ = (θ_*T*_ + δ − μ)/(σ^2^/*N*)^1/2^ + τ_α_(*K*/*v*)^1/2^, *L*_*E*_ = (θ_*T*_ − δ − μ)/(σ^2^/*N*)^1/2^ + τ_1 − α_(*K*/*v*)^1/2^, *Φ* (⋅) is the cumulative density function of the standard normal distribution, and the expectation *E*_*K*_ is taken with respect to the distribution *K*. It is essential to note that the probability *P*{*K* ≥ *κ*_*E*_} ≐ 0 in the subsequent numerical assessments under a wide range of model configurations. This phenomenon is similar to the power computations for the equivalence procedure of two treatment means as noted in Siqueira, et al. [29] and Shieh [30]. Therefore, the exact power appraisal can be numerically approximated by

16 $$ {\varPsi}_{AEQ}=P\;\left\{t\left(v,{\varDelta}_U\right)<{\uptau}_{\upalpha}\right\}-P\left\{t\left(v,{\varDelta}_L\right)<{\uptau}_{1-\upalpha}\right\}, $$

where Δ_*U*_ = (μ – θ_*T*_ – δ)/(σ^2^/*N*)^1/2^ and Δ_*L*_ = (μ – θ_*T*_ + δ)/(σ^2^/*N*)^1/2^.

### Approximate methods

For the purpose of method comparisons, two different approaches for testing normal percentiles are also presented next. To construct confidence intervals of normal percentiles, Bland and Altman [11] and Chakraborti and Li [12] considered simple *t* approximations for the standardized forms of ${\hat{\theta}}_B$ and ${\hat{\theta}}_M,$ respectively. Their methods are extended and examined here for the three types of difference, noninferiority, and equivalence testing.

### The Chakrabort-Li method

In view of the desirable properties of the minimum variance unbiased estimator ${\hat{\theta}}_M,$ Chakraborti and Li [12] suggested an approximate *t* distribution for the standardized quantity of ${\hat{\theta}}_M$:

17 $$ {T}_M=\frac{{\hat{\theta}}_M-\theta}{{\left(m{S}^2/N\right)}^{1/2}}\dot{\sim} t(v). $$

where $m=1+N{z}_p^2\left({c}^2-1\right)$ and *t*(ν) is a *t* distribution with degrees of freedom ν. Note that $Var\left[{\hat{\theta}}_M\right]$ = (*m*σ^2^)/*N* and the denominator of *T*_*M*_ is obtained by a direct substitution of σ^2^ with *S*^2^ in the standard deviation of ${\hat{\theta}}_M$.

The simple formulation of *T*_*M*_ provides an alternative test statistic for judging the magnitude of normal percentiles. For the hypothesis test of difference in terms of H_0_: θ = θ_0_ versus H_1_: θ ≠ θ_0_, the null hypothesis can be rejected at the significance level α if *T*_*M*0_ < *t*_α/2_ or *T*_*M*0_ > *t*_1 − α/2_, or equivalently ∣*T*_*M*0_ ∣  > *t*_1 − α/2_, where

18 $$ {T}_{M0}=\frac{{\hat{\theta}}_M-{\uptheta}_0}{{\left(m{S}^2/N\right)}^{1/2}}, $$

and *t*_α/2_ and *t*_1 − α/2_ are the lower and upper 100(α/2)th quantiles of a *t* distribution *t*(ν) with degrees of freedom ν, respectively. Under the approximate *t* assumption, the corresponding power function can be derived as

19 $$ {\varOmega}_{DI}\left(\varDelta \right)=P\left\{t\left(v,\varDelta \right)<{t}_{\upalpha /2}{m}^{1/2}-{z}_pc{N}^{1/2}\right\}+P\left\{t\left(v,\varDelta \right)>{t}_{1-\upalpha /2}{m}^{1/2}-{z}_pc{N}^{1/2}\right\}. $$

Similarly, the test statistic *T*_*M*0_ can be applied for hypothesis testing of noninferiority of percentiles in terms of H_0_: θ ≤ θ_0_ versus H_1_: θ > θ_0_. The test procedure rejects the null hypothesis at the significance level α if *T*_*M*0_ > *t*_1 − α_ and the associated power function is

20 $$ {\varOmega}_{NI}\left(\varDelta \right)=P\left\{t\left(v,\varDelta \right)>{t}_{1-\upalpha}{m}^{1/2}-{z}_pc{N}^{1/2}\right\}. $$

Moreover, under the hypotheses: H_0_: θ ≥ θ_0_ versus H_1_: θ < θ_0_, the test of noninferiority is rejected if *T*_*M*0_ < *t*_α_ and the corresponding power is given by

21 $$ {\varOmega}_{NI}\left(\varDelta \right)=P\left\{t\left(v,\varDelta \right)<{t}_{\upalpha}{m}^{1/2}-{z}_pc{N}^{1/2}\right\}. $$

For the case of evaluating percentile equivalence with respect to H_0_: θ – θ_*T*_ ≤ −δ or θ – θ_*T*_ ≥ δ versus H_1_: –δ < θ – θ_*T*_ < δ, the null hypothesis is rejected at the significance level α if

22 $$ {T}_{ML}=\frac{{\hat{\theta}}_M-{\uptheta}_T+\updelta}{{\left(m{S}^2/N\right)}^{1/2}}>{t}_{1-\upalpha}\kern0.15em \mathrm{and}\;{T}_{MU}=\frac{{\hat{\theta}}_M-{\uptheta}_T-\updelta}{{\left(m{S}^2/N\right)}^{1/2}}<{t}_{\upalpha}. $$

Accordingly, the power function can be shown as

23 $$ {\varOmega}_{EQ}={E}_K\left[{H}_M\left\{\varPhi \left({U}_M\right)-\varPhi \left({L}_M\right)\right\}\right], $$

where *U*_*M*_ = (θ_*T*_ + δ – μ)/(σ^2^/*N*)^1/2^ + (*t*_α_*m*^1/2^ – *z*_*p*_*cN*^1/2^)(*K*/*v*)^1/2^, *L*_*M*_ = (θ_*T*_ – δ – μ)/(σ^2^/*N*)^1/2^ + (*t*_1 − α_*m*^1/2^ – *z*_*p*_*cN*^1/2^)(*K*/*v*)^1/2^, and *H*_*M*_ = 1 if *K* < κ_*M*_, and *H*_*M*_ = 0 if *K* ≥ κ_*M*_ where ${\kappa}_M=\left( vN{\updelta}^2\right)/\left\{m\upsigma {t}_{1-\upalpha}^2\right\}$. Numerically, the power calculation can be simplified just as Ψ_*AEQ*_ given above:

24 $$ {\varOmega}_{AEQ}=P\left\{t\left(v,{\varDelta}_U\right)<{t}_{\upalpha}{m}^{1/2}-{z}_pc{N}^{1/2}\right\}-P\left\{t\left(v,{\varDelta}_L\right)<{t}_{1-\upalpha}{m}^{1/2}-{z}_pc{N}^{1/2}\right\}. $$

### The Bland-Altman method

Similar to the test procedures based on the minimum variance unbiased estimator, hypothesis testing of normal percentiles can be conducted with the following transformation of ${\hat{\theta}}_B$ in Bland and Altman [11]:

25 $$ {T}_B=\frac{{\hat{\theta}}_B-\theta}{{\left(b{S}^2/N\right)}^{1/2}}\dot{\sim} t(v), $$

where $b=1+{z}_p^2/2$. Specifically, the hypothesis testing of percentile difference in terms of H_0_: θ = θ_0_ versus H_1_: θ ≠ θ_0_ can be rejected at the significance level α if ∣*T*_*B*0_ ∣  > *t*_1 − *α*/2_ where

26 $$ {T}_{B0}=\frac{{\hat{\theta}}_B-{\uptheta}_0}{{\left(b{S}^2/N\right)}^{1/2}}. $$

The associated power function is of the form

27 $$ {\varXi}_{DI}\left(\varDelta \right)=P\left\{t\left(v,\varDelta \right)<{t}_{\upalpha /2}{b}^{1/2}-{z}_p{N}^{1/2}\right\}+P\left\{t\left(v,\varDelta \right)<{t}_{1-\upalpha /2}{b}^{1/2}-{z}_p{N}^{1/2}\right\}. $$

To perform the hypothesis testing of noninferiority with H_0_: θ ≤ θ_0_ versus H_1_: θ > θ_0_, the test rejects the null hypothesis at the significance level α if *T*_*B*0_ > *t*_1 − α_ and the power function is readily obtained as

28 $$ {\varXi}_{NI}\left(\varDelta \right)=P\left\{t\left(v,\varDelta \right)>{t}_{1-\upalpha}{b}^{1/2}-{z}_p{N}^{1/2}\right\}. $$

Likewise, under the hypotheses: H_0_: θ ≥ θ_0_ versus H_1_: θ < θ_0_, the test of noninferiority is rejected if *T*_*B*0_ < *t*_α_ and the corresponding power is expressed as

29 $$ {\varXi}_{NI}\left(\varDelta \right)=P\left\{t\left(v,\varDelta \right)<{t}_{\upalpha}{b}^{1/2}-{z}_p{N}^{1/2}\right\}. $$

Moreover, for the equivalence test of normal percentiles under the hypotheses of H_0_: θ – θ_*T*_ ≤ −δ or θ – θ_*T*_ ≥ δ versus H_1_: –δ < θ – θ_*T*_ < δ, the null hypothesis is rejected at the significance level α if

30 $$ {T}_{BL}=\frac{{\hat{\theta}}_B-{\uptheta}_T+\updelta}{{\left(b{S}^2/N\right)}^{1/2}}>{t}_{1-\upalpha}\kern0.15em \mathrm{and}\;{T}_{BU}=\frac{{\hat{\theta}}_B-{\uptheta}_T-\updelta}{{\left(b{S}^2/N\right)}^{1/2}}<{t}_{\upalpha}. $$

In this case, the power function has the following formulation:

31 $$ {\varXi}_{EQ}={E}_K\left[{H}_B\left\{\varPhi \left({U}_B\right)-\varPhi \left({L}_B\right)\right\}\right], $$

where *U*_*B*_ = (θ_*T*_ + δ – μ)/(σ^2^/*N*)^1/2^ + (*t*_α_*b*^1/2^ – *z*_*p*_*N*^1/2^)(*K*/*v*)^1/2^, *L*_*B*_ = (θ_*T*_ – δ – μ)/(σ^2^/*N*)^1/2^ + (*t*_1 − α_*b*^1/2^ – *z*_*p*_*N*^1/2^)(*K*/*v*)^1/2^, and *H*_*B*_ = 1 if *K* < κ_*B*_, and *H*_*B*_ = 0 if *K* ≥ κ_*B*_ where ${\kappa}_B=\left( vN{\updelta}^2\right)/\left\{b{\upsigma}^2{t}_{1-\upalpha}^2\right\}$. Similar to the other two cases, the power computation can be well approximated by

32 $$ {\varXi}_{AEQ}=P\left\{t\left(v,{\varDelta}_U\right)<{t}_{\upalpha}{b}^{1/2}-{z}_p{N}^{1/2}\right\}-P\left\{t\left(v,{\varDelta}_L\right)<{t}_{1-\upalpha}{b}^{1/2}-{z}_p{N}^{1/2}\right\}. $$

## Results

Numerical investigations are presented next to examine and compare the fundamental features of the exact and approximate test procedures of percentiles with respect to the control of Type I error rate and accuracy of power and sample size computation.

### Tests for difference

For the purpose of illustration, the null $N\left({\upmu}_0,{\upsigma}_0^2\right)$ distribution is set as *N*(0, 1) and two different mean values are considered for the alternative distribution *N*(μ, σ^2^): *N*(0.4, 1) and *N*(0.6, 1). The corresponding percentiles θ_0_ and θ are simplified as θ_0_ = μ_0_ + *z*_*p*_σ_0_ = *z*_*p*_ and θ = μ + *z*_*p*_σ = μ + *z*_*p*_, respectively, with μ = 0.4 and 0.6. For the difference test of percentile in terms of H_0_: θ = θ_0_ versus H_1_: θ ≠ θ_0_, the sample sizes needed to attain the specified power 0.80 for the chosen significance level α = 0.05 are determined by the power functions Ψ_*DI*_, Ω_*DI*_, and Ξ_*DI*_ for *p* = 0.1, …, 0.9. The computed sample sizes for the prescribed three procedures {*T*_*E*0_, *T*_*M*0_, *T*_*B*0_} are summarized in Table 1 for all eighteen combined cases of μ and *p*. It should be noted that the parameter settings are chosen so that the resulting sample sizes have a reasonable magnitude that is often occurred in practice. Moreover, these situations with small and moderate sample sizes are of great importance in the sense that the contending procedures have the obvious potential of yielding distinct outcomes. Monte Carlo simulation studies of 10,000 iterations were conducted for examining the accuracy of the power functions Ψ_*DI*_, Ω_*DI*_, and Ξ_*DI*_. The results reveal that the simulated powers and the attained powers of all three methods agree to the second decimal place for all cases considered here. To save space, the details are not reported.

**Table 1:** The error between simulated alpha and nominal alpha for the difference tests of percentile H_0_: θ = θ_0_ versus H1: θ ≠ θ_0_ with μ_0_ = 0, σ_0_ = 1, σ = 1, and α = 0.05

μ	*p*	Exact approach				Chakraborti-Li method				Bland-Altman method			
		*N*	Lower-tail error	Upper-tail error	Two-tail error	*N*	Lower-tail error	Upper-tail error	Two-tail error	*N*	Lower-tail error	Upper-tail error	Two-tail error
0.4	0.1	109	−0.0020	0.0009	− 0.0011	97	− 0.0101	0.0108	0.0007	95	−0.0108	0.0118	0.0010
	0.2	80	−0.0042	−0.0019	− 0.0061	72	− 0.0113	0.0060	− 0.0053	71	− 0.0121	0.0082	− 0.0039
	0.3	65	−0.0030	−0.0020	− 0.0050	60	− 0.0086	0.0045	− 0.0041	60	− 0.0091	0.0058	− 0.0033
	0.4	57	0.0009	0.0012	0.0021	54	−0.0026	0.0040	0.0014	54	−0.0031	0.0046	0.0015
	0.5	52	0.0024	0.0010	0.0034	52	0.0024	0.0010	0.0034	52	0.0024	0.0010	0.0034
	0.6	50	0.0019	0.0005	0.0024	52	0.0056	−0.0029	0.0027	52	0.0056	−0.0032	0.0024
	0.7	51	0.0020	−0.0004	0.0016	56	0.0093	−0.0083	0.0010	57	0.0101	−0.0088	0.0013
	0.8	58	0.0014	−0.0012	0.0002	66	0.0124	−0.0119	0.0005	66	0.0147	−0.0127	0.0020
	0.9	75	−0.0024	−0.0025	− 0.0049	87	0.0106	−0.0133	− 0.0027	88	0.0133	−0.0138	− 0.0005
0.6	0.1	54	0.0004	0.0012	0.0016	46	−0.0137	0.0155	0.0018	44	−0.0148	0.0181	0.0033
	0.2	40	0.0014	−0.0009	0.0005	34	−0.0105	0.0137	0.0032	33	−0.0111	0.0158	0.0047
	0.3	32	−0.0001	−0.0022	− 0.0023	29	− 0.0094	0.0087	− 0.0007	28	− 0.0103	0.0104	0.0001
	0.4	27	0.0005	−0.0002	0.0003	26	−0.0048	0.0044	−0.0004	25	−0.0053	0.0052	−0.0001
	0.5	24	0.0016	0.0005	0.0021	24	0.0016	0.0005	0.0021	24	0.0016	0.0005	0.0021
	0.6	23	0.0037	0.0017	0.0054	24	0.0105	−0.0037	0.0068	25	0.0117	−0.0041	0.0076
	0.7	22	0.0028	0.0001	0.0029	26	0.0150	−0.0094	0.0056	26	0.0173	−0.0107	0.0066
	0.8	24	0.0014	−0.0012	0.0002	30	0.0183	−0.0155	0.0028	30	0.0209	−0.0159	0.0050
	0.9	31	0.0009	−0.0015	−0.0006	39	0.0201	−0.0174	0.0027	40	0.0248	−0.0182	0.0066

Due to the approximate nature of the *t* distribution associated with the two approximations of Chakraborti and Li [12] and Bland and Altman [11], it is of statistical concern to validate the control of the Type I error rates. Note that the real distribution of the percentile is skewed when sample size is small and *p* deviates considerably from 0.5. This implies that the symmetric *t* approximation of the two test statistics *T*_*M*0_, and *T*_*B*0_ is presumably unsuitable. In other words, the two critical values *t*_α/2_ and *t*_1 − α/2_ are theoretically inaccurate when one-sided rejection probability are evaluated. It is constructive to examine three distinctive Type I errors correspond to the lower-tail, upper-tail, and two-sided rejection regions of the difference tests of percentile.

Accordingly, Monte Carlo simulation studies were also performed to compute the simulated Type I error rates of the exact and approximate test procedures for θ = θ_0_ or μ = 0. The simulated Type I error rate was the proportion of the 10,000 replicates whose test statistic fell in the designated rejection region. In the process, the estimates of the lower-tail and upper-tail rejection rates were computed and summed as the overall or two-sided simulated Type I error rate. The accuracy of the control of Type I error rate can be assessed by the differences between the one-sided and two-sided simulation estimates and the nominal values 0.025 and 0.05, respectively. These differences or errors of the three contending test procedures are also reported in Table 1. It can be readily seen from the results in Table 1 that the all three test methods have excellent control of two-sided Type I error rate. The absolute magnitudes of the errors are less than 0.01 for the investigated mean and percentile configurations.

Moreover, the lower-tail and upper-tail rejection rates of the exact approach are also very close to the nominal levels. But the one-sided Type I error rates of the two approximate methods do not maintain the same accuracy especially for low and high percentiles. Despite the desired performance of the approximate tests in overall Type I error rate, the resulting errors of the lower-tail rejection region tend to be negative for small *p* while those associated with large *p* are constantly positive. In contrast, the upper-tail errors have the exactly opposite outcomes. For the particular case with μ = 0.6 and *p* = 0.9, the induced errors for the approximation of Chakraborti and Li [12] are 0.0201 and − 0.0174 for lower and upper rejection regions, respectively. The corresponding deviated percentages are 0.0201/0.025 = 80.4% and 0.0174/0.025 = 69.6%. To the approximate method of Bland and Altman [11], the lower-tail and upper-tail errors are 0.0248 and − 0.0182 with the deviated percentages 0.0248/0.025 = 99.2% and 0.0182/0.025 = 72.8%, respectively.

### Tests for noninferiority

The underlying characteristics of the exact and approximate methods for the noninferiority test of percentile are also assessed. With the same model formulations in the previous scenario of difference test, the required sample sizes are computed for the hypotheses H_0_: θ ≤ θ_0_ versus H_1_: θ > θ_0_ with the power functions Ψ_*NI*_, Ω_*NI*_, and Ξ_*NI*_. As expected, the result reported in Table 2 is relatively smaller than the counterpart in Table 1 with the identical values of μ and *p*. Moreover, simulation studies were also performed to appraise the actual performance of Type I error for θ = θ_0_ or μ = 0. The errors between the simulated rejection rates and nominal value α = 0.05 are presented in Table 2. Unlike the exact procedure with good control of Type error rate, the two approximate tests do not maintain the required performance. Specifically, when μ = 0.6 and *p* = 0.9, the absolute errors (absolute error percentage) can be as large as 0.0258 (0.0258/0.05 = 51.6%) and 0.0260 (0.0260/0.05 = 52.0%) for *T*_*M*0_ and *T*_*B*0_ of Chakraborti and Li [12] and Bland and Altman [11], respectively. Although the situations improved with increasing sample size as those cases when μ = 0.4, they still suffer some potential deficiency and are outperformed by the exact test.

**Table 2:** The error between simulated alpha and nominal alpha for the non-inferiority tests of percentile H0: θ ≤ θ_0_ versus H1: θ > θ_0_ with μ_0_ = 0, σ_0_ = 1, σ = 1, and α = 0.05

μ	*p*	Exact approach		Chakraborti-Li method		Bland-Altman method	
		*N*	Error	*N*	Error	*N*	Error
0.4	0.1	85	0.0047	77	0.0124	75	0.0168
	0.2	63	0.0017	57	0.0155	56	0.0175
	0.3	51	−0.0011	48	0.0123	47	0.0141
	0.4	44	0.0033	43	0.0046	42	0.0070
	0.5	41	−0.0010	41	0.0004	41	0.0003
	0.6	39	−0.0017	41	−0.0048	41	−0.0077
	0.7	40	−0.0004	44	−0.0112	45	−0.0120
	0.8	46	0.0001	51	−0.0154	52	−0.0183
	0.9	60	−0.0019	68	−0.0170	69	−0.0182
0.6	0.1	42	−0.0027	36	0.0197	35	0.0274
	0.2	31	−0.0042	27	0.0192	26	0.0278
	0.3	25	0.0010	23	0.0119	22	0.0215
	0.4	21	−0.0002	20	0.0052	20	0.0072
	0.5	19	−0.0006	19	−0.0008	19	−0.0014
	0.6	18	−0.0008	19	−0.0086	19	−0.0092
	0.7	18	−0.0013	20	−0.0154	21	−0.0195
	0.8	20	−0.0026	23	−0.0220	24	−0.0252
	0.9	25	−0.0016	31	−0.0258	31	−0.0260

### Tests for equivalence

For the sake of completeness, numerical examination is extended to the equivalence tests of percentile in terms of H_0_: θ – θ_*T*_ ≤ −δ or θ – θ_*T*_ ≥ δ versus H_1_: –δ < θ – θ_*T*_ < δ. In this case, the target percentile and threshold are set as θ_*T*_ = *z*_*p*_ and δ = 0.6, respectively. The alternative normal distribution is selected as *N*(μ, 1) and the associated percentile is θ = μ + *z*_*p*_σ = μ + *z*_*p*_. Then, the power functions Ψ_*AEQ*_, Ω_*AEQ*_, and Ξ_*AEQ*_ are applied to computed the minimum sample sizes required for attaining the nominal power 0.80 at α = 0.05. The resulting sample sizes are listed in Table 3 for μ = 0 and 0.3 and *p* = 0.1, …, 0.9. It was further justified with simulation studies that the power and sample size calculations of the three procedures are all extremely accurate for all eighteen cases reported here. However, power evaluation is valid and informative only when the critical value satisfies the nominal Type I error rate. Additional simulation studies were employed to assess the control of Type I error rates of the equivalence tests {*T*_*EL*_, *T*_*EU*_}, {*T*_*ML*_, *T*_*MU*_}, and {*T*_*BL*_, *T*_*BU*_} for θ = θ_*T*_ – δ or μ = −δ = − 0.6. The errors between the simulated and nominal Type I error rates are presented in Table 3. The assessments show that the two approximate tests {*T*_*ML*_, *T*_*MU*_}, and {*T*_*BL*_, *T*_*BU*_} are not as good as the exact procedure {*T*_*EL*_, *T*_*EU*_}. The deficiency of the two simple *t* distributions is particularly more prominent when the sample size is small and |*p* – 0.5| is large.

**Table 3:** The error between simulated alpha and nominal alpha for the equivalence tests of percentile H_0_: θ – θ_*T*_ ≤ − δ or θ – θ_*T*_ ≥ − δ versus H_1_: - δ < θ - θ_*T*_ < δ with δ = 0.6, θ_*T*_ *=* z_*p*_*,* σ = 1, and α = 0.05

μ	*p*	Exact approach		Chakraborti-Li method		Bland-Altman method	
		*N*	Error	*N*	Error	*N*	Error
0	0.1	47	− 0.0006	45	0.0196	45	0.0257
	0.2	35	0.0003	34	0.0197	34	0.0162
	0.3	30	−0.0015	29	0.0121	29	0.0143
	0.4	27	−0.0039	26	0.0070	26	0.0056
	0.5	26	−0.0006	26	−0.0006	26	−0.0033
	0.6	27	−0.0018	26	−0.0071	26	−0.0111
	0.7	30	−0.0033	29	−0.0144	29	−0.0154
	0.8	35	−0.0047	34	−0.0188	34	−0.0191
	0.9	47	−0.0034	45	−0.0205	45	−0.0212
0.3	0.1	110	−0.0024	121	0.0117	123	0.0205
	0.2	84	0.0000	91	0.0097	92	0.0103
	0.3	73	0.0024	78	0.0063	78	0.0069
	0.4	69	0.0001	72	0.0023	72	0.0043
	0.5	71	0.0015	71	−0.0014	71	0.0012
	0.6	76	0.0008	74	− 0.0081	74	− 0.0069
	0.7	87	−0.0050	82	−0.0068	81	−0.0075
	0.8	106	−0.0041	99	−0.0112	97	−0.0112
	0.9	144	−0.0005	133	−0.0123	131	−0.0152

### An example

To demonstrate the usefulness of the suggested techniques and accompanying programs, a quality control application in pharmaceutical products is exemplified and analyzed with the hypothesis testing and sample size procedures. Suppose a sample of the selected batch of tablets is obtained and tested according to the acceptance sampling plan. Specifically, the dissolution performance is assessed in terms of the percentage of tablets dissolved less than a specified amount at a certain time period.

For illustration, the summary statistics of the dissolution values are $\overline{X}=50.10$ and *S* = 1.31 for *N* = 15. Suppose that the experimenter is interested in the 90th percentile of the distribution of the dissolution quantity. Then, it follows that *z*_0.9_ = 1.2816, *c* = 1.0180, and the minimum variance unbiased estimator is ${\hat{\theta}}_M$ = 51.8091. Using the working settings μ_0_ = 49.3 and σ_0_ = 1.2, the associated 90th percentile value is computed as θ_0_ = μ_0_ + *z*_*p*_σ_0_ = 50.8379. The test statistic *T*_*E*0_ has a value of − 2.1816. For testing the hypotheses of H_0_: θ = 50.8379 versus H_1_: θ ≠ 50.8379, the two critical values or the lower and upper 2.5th quantiles of the noncentral *t* distribution *t*(14, − 4.9636) are τ_0.025_ =  − 8.7695 and τ_0.975_ = − 2.7909, respectively. The null hypothesis is rejected and it implies that the 90th percentile of dissolution amount is not 50.8379 at α = 0.05. Also, it can be shown that the values of the two approximate test statistics for Chakraborti and Li [12] and Bland and Altman [11] are *T*_*M*0_ = 2.0857 and *T*_*B*0_ = 2.0614, respectively, with the critical value *t*_0.975_ = 2.1448. Hence, the two approximate tests suggest that the null hypothesis cannot be rejected for α = 0.05.

Moreover, a noninferiority test can be formed as H_0_: θ ≤ 50.8379 versus H_1_: θ > 50.8379. With a critical value τ_0.05_ = − 3.1072, it indicates that the 90th percentile of dissolution distribution is higher than 50.8379 at the 5% level of significance. In this case, the critical value for the two approximate methods is *t*_0.95_ = 1.7613. Thus, the two approximate tests lead to the same result as the exact procedure. Assume a equivalence test of the 90th percentile is expressed as H_0_: θ – θ_*T*_ ≤ −δ or θ – θ_*T*_ ≥ δ versus H_1_: –δ < θ – θ_*T*_ < δ with θ_*T*_ = 50 + (1.2816)(1.3) = 51.6660 and δ = 1.2. The test statistics are {*T*_*EL*_, *T*_*EU*_} = {− 1.0821, − 8.1776} and the corresponding critical values are {τ_0.95_, τ_0.05_} = {−3.1072, −8.0108}. Thus, there is sufficient evidence to suggest that the 90th percentile is practically 51.6660 for the 5% level of significance. The resulting values for the approximate tests are {*T*_*ML*_, *T*_*MU*_} = {2.8845, − 2.2700} and {*T*_*BL*_, *T*_*BU*_} = {2.8761, − 2.3817}. With the associated critical values *t*_0.95_ = 1.7613 and *t*_0.05_ =  − 1.7613, they also reach the same equivalence outcome. Supplemental computer programs are provided to take advantage of the embedded statistical functions in the interactive matrix language software of Statistical Analysis System (SAS/IML) [31] for performing the prescribed exact test procedures.

For planning future drug dissolution study, sample size calculations should be considered so that the tests have enough power to confirm meaningful magnitude of percentile. It is commonly assumed that typical sources like published findings or expert opinions can offer plausible and reasonable values for the vital characteristics of future study. Hence, the sample statistics of the summary statistics are used as parameter values μ = 50.1 and σ = 1.31. To achieve the nominal power 0.80 with α = 0.05, the constructed SAS/IML programs reveal that the required sample sizes are *N* = 21 and 17 for the test of difference: H_0_: θ = 50.8379 versus H_1_: θ ≠ 50.8379 and the test of noninferiority: H_0_: θ ≤ 50.8379 versus H_1_: θ > 50.8379, respectively. Moreover, for the abovementioned test of equivalence with θ_*T*_ = 51.6660 and δ = 1.2, sample size *N* = 25 is needed to attaining the nominal power 0.80 at α = 0.05. Note that the exemplifying configurations are included in the user specifications of the SAS/IML programs presented in the supplemental files. Accordingly, users can easily modify the input values in these statements to accommodate their own model specifications.

## Discussion

The present investigation generalizes and expands current results in the statistical literature by describing both exact and approximate procedures for the three different percentile tests of difference, noninferiority, and equivalence. The exact approach employs a noncentral *t* distribution, while the approximate techniques follow the familiar *t* distribution as considered in Chakraborti and Li [12] and Bland and Altman [11]. Regarding the two approximate procedures, the results of the conventional tests for difference show that the lower critical value *t*_α/2_ is generally too small for lower normal percentiles and is typically too large for the higher normal percentiles. On the other hand, the upper critical value *t*_1 − α/2_ overestimate and underestimate the correct one for small and large *p*, respectively. Even the overall Type I error is not an issue, it is statistically improper to recommend a two-sided test procedure on the basis of a combination of some noticeable under- and over-sized critical values and rejection regions. Moreover, despite the relatively involved analytic assessments and computational requirements, the comprehensive numerical appraisals show that the exact approach is superior to the approximate methods on the basis of control of Type I errors.

## Conclusion

In view of the conceptual simplicity and context-free feature, percentiles are widely used for determining the relative magnitude and substantial importance of quantitative measurements in all scientific fields. Accordingly, much of the literature has provided the inferential procedures for point and interval estimation of normal percentiles. To extend the applicability of percentile analysis, this article addresses the hypothesis testing problem for the percentiles of a normal distribution. The recommended test procedures and derived power functions are also empirically justified for percentile score assessments and sample size determinations. In order to facilitate data analysis and study planning, specialized computer programs are presented for conducting hypothesis testing and sample size calculation in percentile research.

## Supplementary information

**Additional file 1.** SAS/IML program for conducting percentile test of difference.

**Additional file 2.** SAS/IML program for computing required sample size for percentile test of difference.

**Additional file 3.** SAS/IML program for conducting percentile test of noninferiority.

**Additional file 4.** SAS/IML program for computing required sample size for percentile test of noninferiority.

**Additional file 5.** SAS/IML program for conducting percentile test of equivalence.

**Additional file 6.** SAS/IML program for computing required sample size for percentile test of equivalence.

## Data Availability

All data generated or analysed during this study are included in this published article.

## Abbreviation

SAS/IML: The interactive matrix language software of Statistical Analysis System
