# Supplementary material for: Comparison of alternative approaches for difference, noninferiority, and equivalence testing of normal percentiles
Source: BMC Med Res Methodol. 2020 Mar 13;20:59. doi: 10.1186/s12874-020-00933-z (PMC7071592; doi:10.1186/s12874-020-00933-z)
Supplement: Supplementary file 2 — Additional file 2. SAS/IML program for computing required sample size for percentile test of difference. [file 12874_2020_933_MOESM2_ESM.docx]

Additional file 2

SAS/IML program for computing required sample size for percentile test of difference

PROC IML;

*USER SPECIFICATION PORTION;

*DESIGNATED ALPHA;ALPHA=0.05;

*NOMINAL POWER;POWER=0.8;

*NULL THETA0;THETA0=50.8379;

*MEAN;MU=50.1;

*STANDARD DEVIATION;SIGMA=1.31;

*PERCENTILE;PCT=0.9;

*END OF USER SPECIFICATION PORTION;

ZP=QUANTILE('NORMAL',PCT);SIGSQ=SIGMA##2;THETA=MU+ZP#SIGMA;

PRINT ALPHA PCT ZP[FORMAT=8.4];

PRINT MU SIGMA THETA[FORMAT=8.4] THETA0[FORMAT=8.4];

N=5;

DO UNTIL(EPOWER>POWER);

N=N+1;DF=N-1;

DELTA=(MU-THETA0)/SQRT(SIGSQ/N);

ETL=QUANTILE('T',ALPHA/2,DF,-ZP#SQRT(N));

ETU=QUANTILE('T',1-ALPHA/2,DF,-ZP#SQRT(N));

EPOWER=CDF('T',ETL,DF,DELTA)+SDF('T',ETU,DF,DELTA);

END;

DEPOWER=EPOWER-POWER;

PRINT N EPOWER[FORMAT=8.4] POWER DEPOWER[FORMAT=8.4];

QUIT;
