# Supplementary material for: Comparison of alternative approaches for difference, noninferiority, and equivalence testing of normal percentiles
Source: BMC Med Res Methodol. 2020 Mar 13;20:59. doi: 10.1186/s12874-020-00933-z (PMC7071592; doi:10.1186/s12874-020-00933-z)
Supplement: Supplementary file 3 — Additional file 3. SAS/IML program for conducting percentile test of noninferiority. [file 12874_2020_933_MOESM3_ESM.docx]

Additional file 3

SAS/IML program for conducting percentile test of noninferiority

PROC IML;PRINT 'PRL-NORHT.2NI.EGHT';

*USER SPECIFICATION PORTION;

*DESIGNATED ALPHA;ALPHA=0.05;

*SAMPLE SIZE;N=15;

*SAMPLE MEAN;ME=50.1;

*SAMPLE STANDARD DEVIATION;S=1.31;

*PERCENTILE;PCT=0.9;

*NULL THETA0;THETA0=50.8379;

*END OF USER SPECIFICATION PORTION;

ZP=QUANTILE('NORMAL',PCT);S2=S##2;DF=N-1;

CTL=QUANTILE('T',ALPHA,DF,-ZP#SQRT(N));

CTU=QUANTILE('T',1-ALPHA,DF,-ZP#SQRT(N));

TE0=(ME-THETA0)/SQRT(S2/N);

PRINT ALPHA PCT ZP[FORMAT=8.4] THETA0[FORMAT=8.4] DF;

PRINT ME S N;

PRINT TE0[FORMAT=8.4] CTL[FORMAT=8.4] CTU[FORMAT=8.4];

IF TE0>CTU THEN PRINT "UPPER-TAIL TEST: REJECT H0";

ELSE PRINT "UPPER-TAIL TEST: DON'T REJECT H0";

IF TE0<CTL THEN PRINT "LOWER-TAIL TEST: REJECT H0";

ELSE PRINT "LOWER-TAIL TEST: DON'T REJECT H0";

QUIT;
