# Supplementary material for: Comparison of alternative approaches for difference, noninferiority, and equivalence testing of normal percentiles
Source: BMC Med Res Methodol. 2020 Mar 13;20:59. doi: 10.1186/s12874-020-00933-z (PMC7071592; doi:10.1186/s12874-020-00933-z)
Supplement: Supplementary file 5 — Additional file 5. SAS/IML program for conducting percentile test of equivalence. [file 12874_2020_933_MOESM5_ESM.docx]

Additional file 5

SAS/IML program for conducting percentile test of equivalence

PROC IML;

*USER SPECIFICATION PORTION;

*DESIGNATED ALPHA;ALPHA=0.05;

*SAMPLE SIZE;N=15;

*SAMPLE MEAN;ME=50.1;

*SAMPLE STANDARD DEVIATION;S=1.31;

*PERCENTILE;PCT=0.9;

*THETAT;THETAT=51.6660;

*DELTA;THETAD=1.2;

*END OF USER SPECIFICATION PORTION;

ZP=QUANTILE('NORMAL',PCT);S2=S##2;DF=N-1;

THETAL=THETAT-THETAD;THETAU=THETAT+THETAD;

CTL=QUANTILE('T',ALPHA,DF,-ZP#SQRT(N));

CTU=QUANTILE('T',1-ALPHA,DF,-ZP#SQRT(N));

TEL=(ME-THETAL)/SQRT(S2/N);TEU=(ME-THETAU)/SQRT(S2/N);

T=(TEL>CTU)#(TEU<CTL);

PRINT ALPHA PCT ZP[FORMAT=8.4] THETAT[FORMAT=8.4] THETAD THETAL[FORMAT=8.4] THETAU[FORMAT=8.4];

PRINT ME S N DF;

PRINT TEL[FORMAT=8.4] CTU[FORMAT=8.4] TEU[FORMAT=8.4] CTL[FORMAT=8.4];

IF T=0 THEN PRINT "EQUIVALENCE TEST: DON'T REJECT H0";

ELSE PRINT "EQUIVALENCE TEST: REJECT H0";

QUIT;
