# Supplementary material for: Comparison of alternative approaches for difference, noninferiority, and equivalence testing of normal percentiles
Source: BMC Med Res Methodol. 2020 Mar 13;20:59. doi: 10.1186/s12874-020-00933-z (PMC7071592; doi:10.1186/s12874-020-00933-z)
Supplement: Supplementary file 6 — Additional file 6. SAS/IML program for computing required sample size for percentile test of equivalence. [file 12874_2020_933_MOESM6_ESM.docx]

Additional file 6

SAS/IML program for computing required sample size for percentile test of equivalence

PROC IML;

*USER SPECIFICATION PORTION;

*DESIGNATED ALPHA;ALPHA=0.05;

*NOMINAL POWER;POWER=0.8;

*MEAN;MU=50.1;

*STANDARD DEVIATION;SIGMA=1.31;

*PERCENTILE;PCT=0.9;

*THETAT;THETAT=51.6660;

*DELTA;THETAD=1.2;

*END OF USER SPECIFICATION PORTION;

ZP=QUANTILE('NORMAL',PCT);THETA=MU+ZP#SIGMA;SIGSQ=SIGMA##2;

THETAL=THETAT-THETAD;THETAU=THETAT+THETAD;

PRINT ALPHA POWER PCT ZP[FORMAT=8.4];

PRINT THETAT[FORMAT=8.4] THETAD[FORMAT=8.4] THETAL[FORMAT=8.4] THETAU[FORMAT=8.4];

PRINT MU SIGMA[FORMAT=8.4] THETA[FORMAT=8.4];

NUMINT=1000;LC=NUMINT+1;

COEVECC=({1}||REPEAT({4 2},1,NUMINT/2-1)||{4 1})`;

CL=1E-10;N=5;LOOP=0;

DO UNTIL(EPOWER>POWER|LOOP>1000);

N=N+1;DF=N-1;LOOP=LOOP+1;

ETL=QUANTILE('T',ALPHA,DF,-ZP#SQRT(N));

ETU=QUANTILE('T',1-ALPHA,DF,-ZP#SQRT(N));

KE=(DF#N#(THETAU-THETAL)##2)/(SIGSQ#(ETU-ETL)##2);

INT=KE-CL;INTL=INT/NUMINT;CVEC=CL+(INTL#(0:NUMINT))`;

WCPDF=(INTL/3)#COEVECC#PDF('CHISQ',CVEC,DF);

LEVEC=(THETAL-MU)/SQRT(SIGSQ/N)+ETU#SQRT(CVEC/DF);

UEVEC=(THETAU-MU)/SQRT(SIGSQ/N)+ETL#SQRT(CVEC/DF);

EPOWER=WCPDF`*(CDF('NORMAL',UEVEC)-CDF('NORMAL',LEVEC));

END;

DEPOWER=EPOWER-POWER;

PRINT N EPOWER[FORMAT=8.4] POWER DEPOWER[FORMAT=8.4];

QUIT;
